# Variations of pterygium prevalence by age, gender and geographic characteristics in China: A systematic review and meta-analysis

**DOI:** 10.1371/journal.pone.0174587

**Published:** 2017-03-29

**Authors:** Peige Song, Xinlei Chang, Manli Wang, Lin An

**Affiliations:** 1 Department of Child, Adolescent and Women's Health, School of Public Health, Peking University, Beijing, China; 2 Centre for Population Health Sciences, University of Edinburgh, Edinburgh, Scotland, United Kingdom; Soochow University Medical College, CHINA

## Abstract

**Background:**

Pterygium is a common chronic ophthalmic condition, which may result in significant visual morbidity or lead to blindness in extreme cases. The prevalence of pterygium in China has not been reported at the sub-national level.

**Methods:**

In this study, we conducted a systematic review and meta-analysis to estimate the prevalence of pterygium in China. China National Knowledge Infrastructure (CNKI), Wanfang, Chinese Biomedicine Literature Database (CBM-SinoMed), PubMed, Embase and Medline were searched before September 2016. We performed a multilevel mixed-effect meta-regression based on the included studies, our results showed that age, gender and latitude were significantly associated with pterygium prevalence. Based on the final model, the age and gender-specific prevalence of pterygium in 31 Chinese provinces (except Hongkong, Macau and Taiwan) and the whole country was generated.

**Results:**

In 2010, the overall prevalence of pterygium in Chinese people aged 15–84 years was 9.84% (95% CI: 6.72–14.14), and the number of pterygium cases in China was 108.65 million (95% CI: 74.23–156.13).

**Conclusions:**

In conclusion, the prevalence of pterygium in Chinese population in 2010 was estimated at both the national and provincial levels. The higher burden of pterygium across the country calls for efforts to advocate public health education encouraging people to take appropriate protective measures.

## Introduction

Pterygium, a wing-shaped fibrovascular growth of the bulbar conjunctiva, is a common chronic ophthalmic condition [1, 2]. Although pterygium is generally regarded as a benign and cosmetic concern, without proper treatment, it may result in significant visual morbidity or even potentially blindness in extreme stages [3, 4]. The aetiology and pathogenesis of pterygium remain uncertain [5]. Previous studies suggest that older age, male gender and outdoor occupation may be risk factors for the presence of pterygium [1, 5–7]. In addition, epidemiology surveys indicate that tropical areas tend to show higher rates of pterygium, this geographical variation may reveal a positive relationship between ultraviolet radiation exposure and the presence of pterygium [8].

In China, the biggest developing country with large geographical variation by latitude and longitude, the reported prevalence of pterygium varied widely from 2.9% for people aged 40 years and above in the north (rural Beijing) to 33.0% for people aged 50 years and above in the south (rural Guangdong) [7–10]. Although the latest meta-analysis of worldwide pterygium prevalence conducted by L Liu, et al. has revealed a pooled pterygium prevalence of 9.9% in the Chinese population, the deficiency of Chinese literature limited their ability to explore the geographical variation of pterygium prevalence in-depth within the country [8]. China's bibliographic databases have long been regarded as an unexplored resource for understanding the epidemiology of diseases in China [11–14], in this study, we conducted a systematic review of previous population-based studies on the prevalence of pterygium in China and investigated the differences in prevalence by age, gender and geographic factors.

## Methods

### Search strategy and selection criteria

We conducted the search to identify all papers published between January 1990 and September 2016. The searched databases included three Chinese bibliographic databases and three English bibliographic databases, namely, China National Knowledge Infrastructure (CNKI), Wanfang, Chinese Biomedicine Literature Database (CBM-SinoMed), PubMed, Embase and Medline. A combination of the following search terms was applied: “incidence” or “prevalence” or “morbidity” or “mortality” or “epidemiology”, combined with “pterygium” and “China or Chinese”. Snowball searching of reference lists was also conducted to further identify studies of interest.

This systematic review followed the guidelines of the Preferred Reporting Items for Systematic reviews and Meta–Analyses (PRISMA) guidelines (S1 Table) [15]. No protocol for this systematic review was pre-registered. All citations were reviewed by two researchers (XXC and MLW) independently. All uncertainties were resolved by consensus. The inclusion criteria were: (i) population-based study of pterygium in China; (ii) studies conducted to examine the epidemiology of pterygium; (iii) studies with clear assessment methods and diagnose of pterygium. Duplicate publications of the same study were compared and the one with more details was kept. In addition, studies that were conducted in unrepresentative populations were excluded, e.g., diabetic population.

### Data extraction

Two researchers (XXC and MLW) independently extracted data using piloted standardised data extraction form, any disagreements were resolved by reviewing and group discussion. The key information included: authors, publication year, study site, study year, study design, age, gender, and the number of participants and pterygium cases. The latitude and longitude information of the survey areas, as reported in each study, was obtained using Google Maps GPS coordinates (http://www.gps-coordinates.net/). For each area, the average annual insolation data (i.e., the amount of solar radiation incident on the surface of the earth) on the horizontal surface, expressed in kWh/m^2^/day, was obtained from the National Aeronautics and Space Administration (NASA) Atmospheric Science Data Centre (http://eosweb.larc.nasa.gov/sse/). The presence of pterygium was defined as an extension of the conjunctiva onto the clear cornea.

### Statistical analysis

Study year was calculated by taking the median date within the study investigation period. Based on the average difference of the study year and published year in papers with available data, three years were subtracted from the published year to impute the missing data of study year. For studies with censoring age groups, e.g. older than 80 years, the missing age band was taken as the same width as other age groups in the same paper, the midpoint of the age range was adopted as the age variable for analysis. Some studies contributed only one data point, whereas others contributed several different data points by age, gender or setting groups. We defined gender and setting as mixed when only overall estimates of the pterygium prevalence were reported with no further information to stratify results by gender and setting.

Firstly, the variances of the raw prevalence estimates were stabilised by using logit transformation. To assess the heterogeneity of the prevalence across studies, the Cochran's Q statistic and *I*^2^ index were calculated [16, 17]. A p-value<0.05 indicates heterogeneity in the effect size between studies in Q statistic, and *I*^2^ represents the percentage of the total variability due to heterogeneity rather than chance, where a value of 0% indicates no observed heterogeneity and values of 25%, 50% and 75% reflect low, moderate and high heterogeneity, respectively. Due to the high heterogeneity across studies (*I*^2^ >75%), the overall prevalence was pooled based on the random-effect (DerSimonian and Laird method) meta-analysis [16]. Forest plots were also generated to illustrate the prevalence with corresponding 95% Confidence Intervals (CIs) for each study and the overall random-effects pooled estimate.

Moreover, to discover relevant moderators that could account for the variance in the overall prevalence rate and take the hierarchical structure and non-independence of data within the same study into consideration, we performed a multilevel mixed-effect meta-regression based all the data points provided by the included studies [18, 19]. The logit transformation of prevalence data was adopted in the regression [20, 21]. Given that:

$$prevalence=p=\frac{number\;of\;cases}{sample\;size}$$

Then, using the logit transformation:

$$logit(p)=\log_{e}(\frac{p}{1-p})=\log_{e}\left(odds\right)=\alpha +\beta_{1}*x_{1}+\beta_{1}*x_{1}+\cdots \beta_{n}*x_{n}$$

Estimates were back transformed and expressed as conventional prevalence. The relevant moderators included age, gender, study setting, study year, latitude, longitude and annual insolation. Moderators that had a p-value lower than 0.05 in the univariate analyses were included in the subsequent multivariate regression analysis.

Finally, the age and gender-specific prevalence of pterygium in 31 Chinese provinces (except Hongkong, Macau and Taiwan) was generated based on the final model. By multiplying the corresponding age and gender-specific populations in each province for the year 2010, obtained from the 6^th^ national census of China [22], the numbers of people with pterygium in 31 provinces were derived. The prevalence of pterygium was then calculated by dividing the total number of pterygium cases by the total population in each province and nationally. The final provincial and national gender-specific prevalence of pterygium were visualised on the China map, which was obtained as shapefile from the Global Administrative Areas (GADM) database (GADM, 2015, version 2.0; www.gadm.org).

All p-values were 2-sided, and p < 0.05 was considered to indicate statistical significance. All analyses were undertaken with R, version 3.3.0 (R Foundation for Statistical Computing, Vienna, Austria), maps were drawn using ArcMap version 10.1 (Environmental Systems Research Institute, Redlands, CA).

## Results

### Study identification and selection

**Fig 1:**
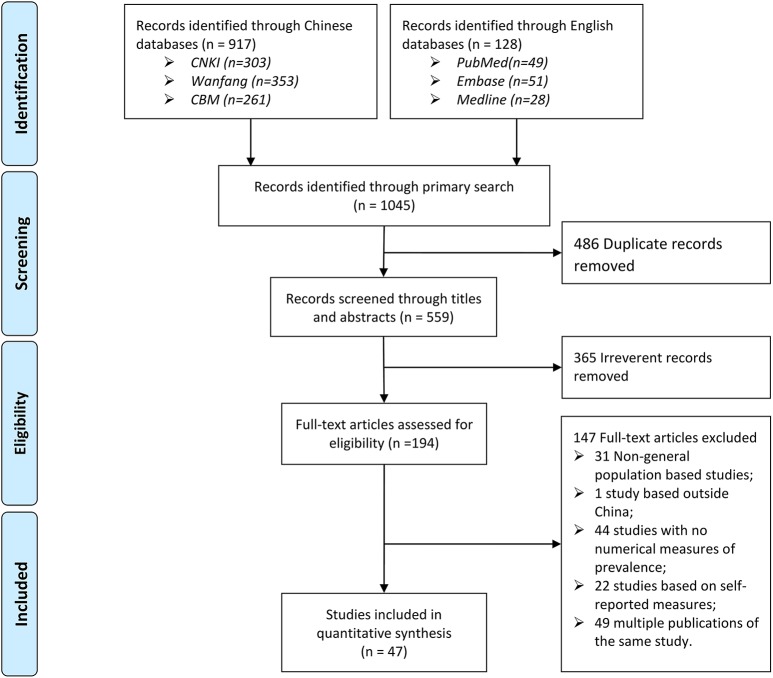
PRISMA flow chart.

The primary search returned 1045 citations. After removing 486 duplicates and 365 apparently irrelevant citations by title and abstract review, 194 articles were reviewed to assess their eligibility at the full-text level. Finally, 47 studies were included in the final quantitative synthesis (Fig 1).

### Study characteristics

Table 1 summarises the main characteristics of the studies, and the detailed information was listed in S2 Table. A total of 47 studies with 406995 participants were included. Of these, 31195 participants were diagnosed with pterygium. Most studies were published after 2005 (n = 43, 91.5%), and mainly from the rural area (n = 31, 66.0%), whereas only 4 studies provided information on prevalence in urban areas. Most studies provided prevalence for both men and women (n = 40, 85.1%). A total of 39 studies (83.0%) consisted of 10,000 participants or less, while 8 studies (17.0%) consisted of at least 10,000 participants. All studies had clear pterygium assessment, and the majority (n = 38, 80.9%) of which used slit lamp examination method.

**Table 1:** Main characteristics of the retained studies (n = 47).

Characteristics of study	Number of studies (%)
**Year published**	
1990–1999	2 (4.3)
2000–2004	2 (4.3)
2005–2009	14 (29.8)
2010–2014	21 (44.7)
2015–2016	8 (17.0)
**Setting**	
Urban	1 (2.1)
Rural	31 (66.0)
Both	3 (6.4)
Mixed	12 (25.5)
**Gender**	
Both	40 (85.1)
Mixed	7 (14.9)
**Sample size**	
301–2000	6 (12.8)
2001–5000	20 (42.6)
5001–10000	13 (27.7)
10001–20000	4 (8.5)
20001–102000	4 (8.5)
**Assessment tool**	
Cornea examination or photography	3 (6.4)
External ocular and fundus photography	4 (8.5)
Flashlight	1 (2.1)
Flashlight and slit lamp examination	10 (21.3)
Slit lamp examination	28 (59.6)
General eye examination	1 (2.1)

### Meta-analysis and meta-regression

The meta-analysis revealed significantly high heterogeneity across studies (*I*^2^ = 99.8%, p<0.001), the overall pooled prevalence of pterygium in the included studies was 10.31% (95% CI = 7.96–13.26), the corresponding forest is shown in S1 Fig. In the univariate meta-regression analysis (Table 2), age, gender and latitude were significantly associated with the pterygium prevalence, all these three moderators were then included in the final multivariate regression model (Table 2).

**Table 2:** Multilevel univariate and multivariate meta-regression models of the various factors related to the prevalence of pterygium.

Moderator	Number of studies	β	95% CI	P value
**Univariate meta regression**				
Intercept	47	-2.008	[-2.346]-[-1.670]	<0.001
Age	47	0.042	0.040–0.043	<0.001
Gender-Male^$^	40	0.115	0.083–0.147	<0.001
Latitude	47	-0.072	[-0.114]-[-0.030]	<0.001
**Multivariate meta regression**^**#**^				
Intercept	40	-2.069	[-3.735]-[-0.403]	0.015
Age	40	0.042	0.040–0.043	<0.001
Gender-Male	40	0.105	0.073–0.137	<0.001
Latitude	40	-0.069	[-0.117]-[-0.022]	0.004

^$^ the estimate of gender effect was based on studies that provided pterygium prevalence for both males and females.

^#^ the multivariate model was based on studies that provided pterygium prevalence for both males and females; coefficients represent log odds ratios (ORs).

### Estimates of pterygium prevalence and cases

**Fig 2:**
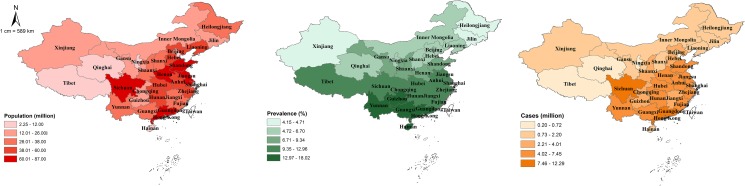
The geographical distribution of population, pterygium prevalence and number of people with pterygium in China in 2010. (a) number of population; (b) overall prevalence of pterygium; (c) number of people with pterygium. The map was created by PGS using ArcMap version 10.1 (Environmental Systems Research Institute, Redlands, CA).

Based on the final model, the age and gender-specific prevalence of pterygium was estimated for people aged 15–84 years in 31 provinces, the overall prevalence and cases of pterygium across the whole China are shown in Fig 2. In 2010, Tibet was the least populous province whereas Guangdong owed the most population (2.26 million vs. 86.11 million). The overall prevalence of pterygium was the lowest in Heilongjiang and the highest in Hainan (4.16% vs. 18.02%), which showed an obvious increasing trend with latitudes. Consistent with the distribution of population, the total number of people living with pterygium was the lowest in Tibet and highest in Guangdong (0.21 million vs. 12.29 million).

**Fig 3:**
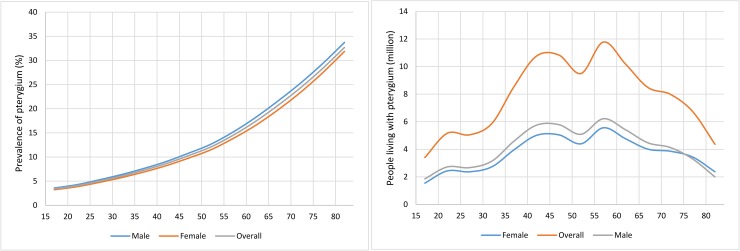
Gender- and age-specific prevalence of pterygium and numbers of people living with pterygium in 2010.

Table 3 and Fig 3 list the age-specific prevalence of pterygium in 2010 for the whole country. In 2010, the prevalence of pterygium ranged from 3.41% (95% CI: 2.19–5.33) in adolescents aged 15–19 years to 32.68% (95% CI: 24.23–42.22) in older people aged 80–84 years, the overall prevalence of pterygium in people aged 15–84 years was 9.84% (95% CI: 6.72–14.14). The prevalence of pterygium showed an increasing trend with the increase of age in both men and women. The number of pterygium cases in China was 108.65 million (95% CI: 74.23–156.13) in people aged 15–84 years. Although the prevalence of pterygium increased with the increase of age, the number of people with pterygium didn’t show the same trend because of the demographic structure. Most pterygium cases (11.78 million [95% CI: 8.13–16.67]) were in people aged 55–59 years.

**Table 3:** Estimated gender- and age-specific prevalence of pterygium and number of people living with pterygium in China in 2010.

	Prevalence of pterygium (%, 95% CI)			People living with pterygium (million, 95% CI)		
	Male	Female	Overall	Male	Female	Overall
15–19 years	3.58	3.22	3.41	1.86	1.55	3.41
	(2.30–5.60)	(2.07–5.03)	(2.19–5.33)	(1.19–2.91)	(0.99–2.42)	(2.19–5.32)
20–24 years	4.26	3.84	4.05	2.72	2.43	5.16
	(2.75–6.58)	(2.48–5.93)	(2.62–6.26)	(1.76–4.21)	(1.58–3.76)	(3.33–7.97)
25–29 years	5.26	4.73	5.00	2.67	2.37	5.05
	(3.38–8.14)	(3.06–7.31)	(3.22–7.73)	(1.72–4.14)	(1.53–3.67)	(3.25–7.80)
30–34 years	6.35	5.73	6.04	3.14	2.73	5.87
	(4.12–9.71)	(3.73–8.75)	(3.93–9.24)	(2.04–4.81)	(1.77–4.17)	(3.81–8.97)
35–39 years	7.61	6.88	7.26	4.60	3.97	8.56
	(5.01–11.45)	(4.53–10.35)	(4.78–10.92)	(3.02–6.92)	(2.61–5.97)	(5.64–12.88)
40–44 years	9.04	8.19	8.62	5.75	5.01	10.76
	(6.02–13.38)	(5.47–12.13)	(5.75–12.77)	(3.83–8.51)	(3.34–7.42)	(7.17–15.93)
45–49 years	10.76	9.75	10.26	5.79	5.05	10.84
	(7.23–15.72)	(6.55–14.27)	(6.90–15.01)	(3.89–8.46)	(3.40–7.40)	(7.28–15.85)
50–54 years	12.62	11.47	12.06	5.09	4.40	9.50
	(8.58–18.17)	(7.78–16.59)	(8.19–17.40)	(3.46–7.33)	(2.99–6.37)	(6.45–13.70)
55–59 years	15.14	13.81	14.48	6.22	5.56	11.78
	(10.47–21.37)	(9.52–19.6)	(10.00–20.50)	(4.30–8.78)	(3.83–7.89)	(8.13–16.67)
60–64 years	18.13	16.50	17.33	5.41	4.76	10.17
	(12.70–25.15)	(11.51–23.04)	(12.12–24.11)	(3.79–7.50)	(3.32–6.64)	(7.11–14.15)
65–69 years	21.53	19.69	20.62	4.47	4.01	8.48
	(15.28–29.34)	(13.9–27.06)	(14.60–28.21)	(3.17–6.09)	(2.83–5.51)	(6.00–11.60)
70–74 years	25.18	23.27	24.22	4.13	3.86	7.99
	(18.09–33.76)	(16.61–31.47)	(17.34–32.61)	(2.97–5.54)	(2.75–5.21)	(5.72–10.75)
75–79 years	29.24	27.34	28.24	3.30	3.44	6.74
	(21.38–38.40)	(19.85–36.19)	(20.57–37.24)	(2.41–4.33)	(2.50–4.55)	(4.91–8.88)
80–84 years	33.72	31.85	32.68	2.00	2.37	4.37
	(25.09–43.40)	(23.55–41.29)	(24.23–42.22)	(1.48–2.57)	(1.76–3.08)	(3.24–5.65)
15–84 years	10.21	9.46	9.84	57.15	51.50	108.65
	(6.97–14.67)	(6.47–13.6)	(6.72–14.14)	(39.03–82.09)	(35.20–74.04)	(74.23–156.13)

## Discussion

The prevalence of pterygium varies widely as reported in different studies, the high variation might be partly explained by varying risk factors, such as sun exposure, gender and ethnic groups [1, 3, 5–8, 10]. Although the large magnitude of population and geographic scale in China limit large-scale public health data collection on ocular diseases. The sufficient information of local community-based surveys in Chinese bibliographic databases allows a model-based pathway to explore the ocular disease burden in China. Our study provides a comprehensive overview of pterygium distribution in China at both the national and provincial levels. Previous global estimates of pterygium prevalence showed a pooled prevalence of 10.2% (95% CI 6.3% to 16.1%) in the general population [8], this high burden has also been witnessed in our analysis for China, where the prevalence of pterygium has been estimated as 9.84% (95% CI: 6.72–14.14) at the national level. Prevalence estimates also indicated a higher burden in provinces with lower latitude, older people and men in particular. Moreover, the large number of people living with pterygium also strongly highlighted the need for pterygium prevention and treatment in China.

Previous studies revealed the positive relationship between ultraviolet exposure and the presence of pterygium, which suggests a higher prevalence of pterygium in the people living near equator [6, 8]. To shed light on the possible factors explaining this result, we incorporated all available geographic information and other demographic variables, and the association with the risk of pterygium by using a meta-regression approach. Our analysis showed that the prevalence of pterygium was significantly related to latitude, whereas no difference was revealed for people living in different longitudes, this finding was in line with the previous global meta-analysis [8]. However, the increase of annual insolation was not found as a risk factor, this finding was in contrast with the positive relationship between ophthalmic disorders and exposure to sunlight [23, 24]. There are two possible reasons contributing to this phenomenon: First, annual insolation data was averaged over a 22-year period (July 1983—June 2005), which may represent a considerable time-lag [25, 26]. Second, the relation between insolation and the prevalence of AMD may not be a monotone function. Although this interesting relation was not able to be explored because of data availability in our study, the negative relation between latitude and pterygium prevalence serves as a very good incentive to discover the relation between insolation and pterygium prevalence in future Chinese pterygium epidemiological studies.

In the present study, age was revealed as a significant risk factor for pterygium, this has also been previously proven in many other studies [3, 5, 8, 27], the difference may be related to increasing vulnerability of ultraviolet radiation in older people [4, 28]. In common with other surveys of pterygium [1, 8, 29], the presence of pterygium in China was also more frequently in men than in women. The may reflect variations of outdoor exposure and occupations between different genders. However, no sufficient information about job history or time of sun exposure can be gathered to support this hypothesis, further studies are still needed to explore this relation in general Chinese population.

It is still worth pointing out the limitations. First, despite the strict inclusion and exclusion criteria applied, various studies have used different instruments to examine and diagnose pterygium, significant heterogeneity existed between all of the included studies. Although we made the best effort to include all relevant studies on pterygium prevalence in China, all of the included studies were published results, the deficiency of unpublished results in the analysis may bring bias in our estimates. In addition, our meta-regression model only included study-level moderators, other important individual-level variations, such as occupations, time of sun exposure, and the habit of wearing sunglasses were not explored [1, 4, 30]. Second, our estimates on the number of pterygium cases were only based on the 2010 Chinese census because of the availability of demographic data, this may represent a considerable time-lag for assessing the most up-to-date burden of pterygium in China. Third, in the final meta-regression model, the setting (urban vs. rural) was not a significant moderator, given the fact that most of the included studies were conducted in rural areas, our results may present an under- or over-estimation of the pterygium prevalence if the inherent urban-rural difference does exist. Fourth, the studies did not come from across the country, although the geographic variation of pterygium prevalence has been demonstrated in our analysis, the ability to generate estimates of provincial prevalence may be limited. Furthermore, the provincial estimates of pterygium prevalence were based on the assumption that pterygium prevalence within individual provinces was homogeneous, which was too rough for huge provinces where the variation of geographic factors and occupations may exist within the provinces.

In conclusion, the prevalence of pterygium was 9.84% in Chinese people aged 15–84 years in 2010, which is similar to the global prevalence of pterygium. Older age, male gender and higher latitude were all associated with higher risk of pterygium. Future research is still needed in China to explore the variations of pterygium burden in groups with specific individual behaviours. The higher burden of pterygium across the country calls for efforts to advocate public health education encouraging people to take appropriate protective measures, such as wearing sunglasses or hats in outdoor environments. Especially for individuals living in southern China where pterygium prevalence is generally much higher.

## Supporting information

S1 Fig Forest plot displaying the pooled prevalence of pterygium in population-based studies. (TIF)

S1 Table PRISMA checklist. (DOC)

S2 Table Characteristics of the included studies. (DOCX)
