# Supplementary figures and images for: Variations of pterygium prevalence by age, gender and geographic characteristics in China: A systematic review and meta-analysis
Source: PLoS One. 2017 Mar 29;12(3):e0174587. doi: 10.1371/journal.pone.0174587 (PMC5371366; doi:10.1371/journal.pone.0174587)

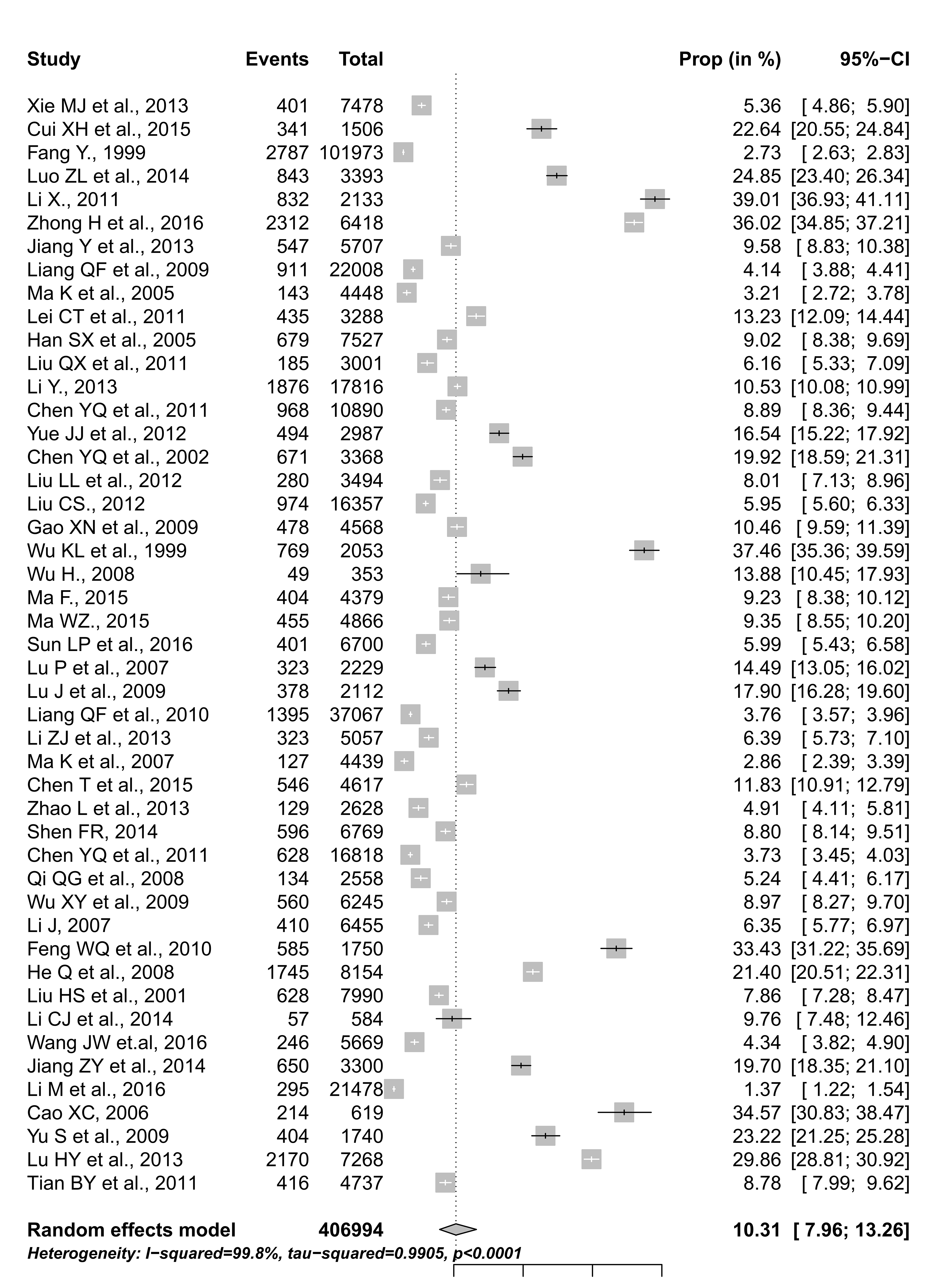

Supplement: S1 Fig — (TIF) [file pone.0174587.s001.tif]
