# Supplementary material for: Variations of pterygium prevalence by age, gender and geographic characteristics in China: A systematic review and meta-analysis
Source: PLoS One. 2017 Mar 29;12(3):e0174587. doi: 10.1371/journal.pone.0174587 (PMC5371366; doi:10.1371/journal.pone.0174587)
Supplement: S2 Table — (DOCX) [file pone.0174587.s003.docx]

**Table S2. Characteristics of the included studies.**

| Author | Year Published | Province | Study year | Sampling | Assessment | Setting | Gender | Age range | Sample size | Cases | Prevalence (%) |
| --- | --- | --- | --- | --- | --- | --- | --- | --- | --- | --- | --- |
| Fang Y | 1999 | Anhui | 1999 | Randomized, clustered sampling | slit lamp examination | Mixed | M/F | 10-89 | 101973 | 2787 | 2.73 |
| Ma K et al. | 2005 | Beijing | 2001 | Randomized, clustered sampling | external ocular and fundus photography | Mixed | M/F | 40-89 | 4448 | 143 | 3.21 |
| Ma K et al. | 2007 | Beijing | 2001 | NS | corneal photography | Mixed | M/F | 40-101 | 4439 | 128 | 2.88 |
| Liang QF et al. | 2009 | Beijing | 2008 | NS | external ocular and fundus photography | R | M/F | 55-85 | 22008 | 911 | 4.14 |
| Liang QF et al. | 2010 | Beijing | 2009 | NS | external ocular and fundus photography | R | M/F | 55-85 | 37067 | 1395 | 3.76 |
| Zhao L et al. | 2013 | Beijing | 2011 | NS | slit lamp examination | Mixed | M/F | 40-79 | 2628 | 129 | 4.91 |
| Ma F | 2015 | Gansu | 2013 | Randomized, clustered sampling | slit lamp examination | R | M/F | 35-74 | 4379 | 404 | 9.23 |
| Wu KL et al. | 1999 | Guangdong | 1997 | Randomized, clustered sampling | flashlight and slit lamp examination | R | M/F | 50-99 | 2053 | 769 | 37.46 |
| Yue JJ et al. | 2012 | Guangdong | 2008 | Randomized, clustered sampling | slit lamp examination | Mixed | M/F | 35-99 | 2987 | 494 | 16.54 |
| Luo ZL et al. | 2014 | Guangdong | 2013 | Randomized, clustered sampling | slit lamp examination | Mixed | M/F | 40-79 | 3393 | 843 | 24.85 |
| Jiang ZY et al. | 2014 | Guizhou | 2011 | clustered random sampling | flashlight and slit lamp examination | R | M/F | 50-95 | 3300 | 650 | 19.70 |
| Liu HS et al. | 2001 | Hainan | 1999 | clustered random sampling | general eye examination | R | M/F | 12-88 | 7990 | 628 | 7.86 |
| Li CJ et al. | 2014 | Hainan | 2013 | NS | slit lamp examination | Mixed | Mixed | 60-85 | 584 | 57 | 9.76 |
| Li J | 2007 | Hebei | 2006 | proportional stratified clustered random sampling | slit lamp examination | R | M/F | 40-89 | 6455 | 410 | 6.35 |
| Liu LL et al. | 2012 | Hebei | 2011 | Randomized, clustered sampling | external ocular and fundus photography | R | M/F | 50-93 | 3494 | 280 | 8.01 |
| Liu CS | 2012 | Hebei | 2009 | Randomized, clustered sampling | slit lamp examination | Mixed | M/F | 16-80 | 16357 | 974 | 5.95 |
| Sun LP et al. | 2016 | Hebei | 2006 | Randomized, clustered sampling | slit lamp examination | R | M/F | 30-79 | 6701 | 401 | 5.98 |
| Li ZJ et al. | 2013 | Heilongjiang | 2006 | stratified, clustered randomized sampling | slit lamp examination | R | Mixed | 50-96 | 5057 | 323 | 6.39 |
| Wang JW et.al | 2016 | Heilongjiang | 2013 | clustered random sampling | slit lamp examination | R | M/F | 59-91 | 5669 | 246 | 4.34 |
| Chen YQ et al. | 2011 | Hubei | 2008 | clustered random sampling | slit lamp examination | U/R | Mixed | <20->70 | 16818 | 628 | 3.73 |
| Qi QG et al. | 2008 | Inner Mongolia | 2005 | stratified clustered random sampling | slit lamp examination | U/R | M/F | 10-79 | 2558 | 134 | 5.24 |
| Jiang Y et al. | 2013 | Inner Mongolia | 2010 | Randomized, clustered sampling | slit lamp examination | Mixed | M/F | 40-89 | 5707 | 547 | 9.58 |
| Wu XY et al. | 2009 | Jiangsu | 2007 | clustered random sampling | flashlight and slit lamp examination | R | Mixed | 40-89 | 6245 | 560 | 8.97 |
| Lu HY et al. | 2013 | Jiangxi | 2012 | clustered random sampling | flashlight and slit lamp examination | R | M/F | 40-92 | 7268 | 2170 | 29.86 |
| Liu QX et al. | 2011 | Ningxia | 2008 | Randomized, clustered sampling | cornea examination | Mixed | M/F | 6-75 | 3001 | 185 | 6.16 |
| Lu P et al. | 2007 | Qinghai | 2006 | stratified,clustered randomized sampling | slit lamp examination | R | M/F | 40-89 | 2229 | 323 | 14.49 |
| Lu J et al. | 2009 | Qinghai | 2006 | stratified clustered random sampling | slit lamp examination | R | M/F | 40-89 | 2112 | 378 | 17.90 |
| Tian BY et al. | 2011 | Shaanxi | 2003 | clustered random sampling | slit lamp examination | R | M/F | 20-69 | 4737 | 416 | 8.78 |
| Gao XN et al. | 2009 | Shandong | 2007 | Randomized, clustered sampling | flashlight | R | M/F | 40-79 | 4568 | 478 | 10.46 |
| Li Y | 2013 | Shandong | 2008 | Randomized, clustered sampling | slit lamp examination | R | M/F | 50-101 | 17816 | 1876 | 10.53 |
| Ma WZ | 2015 | Shandong | 2008 | Randomized, clustered sampling | slit lamp examination | Mixed | M/F | 50-89 | 4866 | 455 | 9.35 |
| Li M et al. | 2016 | Shandong | 2009 | clustered random sampling | flashlight and slit lamp examination | R | M/F | 31-80 | 21478 | 295 | 1.37 |
| Cui XH et al. | 2015 | Shanghai | 2010 | Randomized, clustered sampling | slit lamp examination | R | M/F | 50-89 | 1506 | 341 | 22.64 |
| Shen FR | 2014 | Shanxi | 2011 | NS | corneal photography | R | M/F | 50-89 | 6769 | 596 | 8.80 |
| Lei CT et al. | 2011 | Sichuan | 2008 | Randomized, clustered sampling | slit lamp examination | R | M/F | 50-90 | 3288 | 435 | 13.23 |
| Xie MJ et al. | 2013 | Sichuan | 2011 | Randomized, two stages sampling | slit lamp examination | U | M/F | 40-89 | 7478 | 401 | 5.36 |
| Han SX et al. | 2005 | Tianjin | 2004 | Randomized, clustered sampling | flashlight and slit lamp examination | R | M/F | 40-89 | 7527 | 679 | 9.02 |
| Chen YQ et al. | 2002 | Xinjiang | 2001 | Randomized, clustered sampling | flashlight and slit lamp examination | R | M/F | 50-93 | 3368 | 671 | 19.92 |
| Wu H | 2008 | Xinjiang | 1996 | Randomized, stratified sampling | slit lamp examination | R | M/F | 6-99 | 353 | 49 | 13.88 |
| Chen YQ et al. | 2011 | Xinjiang | 2000 | Randomized, clustered sampling | slit lamp examination | R | M/F | 11-94 | 10890 | 968 | 8.89 |
| Chen T et al. | 2015 | Xinjiang | 2012 | multistage, stratified random sampling | slit lamp examination | Mixed | Mixed | 30-80 | 4617 | 546 | 11.83 |
| Cao XC | 2006 | Yunnan | 2005 | clustered random sampling | slit lamp examination | R | Mixed | 1-58 | 619 | 214 | 34.57 |
| Li X | 2011 | Yunnan | 2010 | Randomized, clustered sampling | slit lamp examination | R | M/F | 50-92 | 2133 | 832 | 39.01 |
| Zhong H et al. | 2016 | Yunnan | 2012 | Randomized, clustered sampling | slit lamp examination | R | Mixed | 50-92 | 6418 | 2312 | 36.02 |
| He Q et al. | 2008 | Zhejiang | 2007 | randomized sampling | flashlight and slit lamp examination | R | M/F | 18-80 | 8154 | 1745 | 21.40 |
| Yu S et al. | 2009 | Zhejiang | 2007 | clustered random sampling | flashlight and slit lamp examination | U/R | M/F | 41-80 | 1740 | 404 | 23.22 |
| Feng WQ et al. | 2010 | Zhejiang | 2009 | clustered random sampling | flashlight and slit lamp examination | R | M/F | 55-84 | 1750 | 585 | 33.43 |

U is short for Urban, R for Rural, U/R means the study provided prevalence estimates for both; M is short for Male, F for Female, M/F means the study provided prevalence estimates for both.
